# Mathematical modelling of the internal circulation anaerobic reactor by Anaerobic Digestion Model No. 1, simultaneously combined with hydrodynamics

**DOI:** 10.1038/s41598-019-42755-0

**Published:** 2019-04-18

**Authors:** Yifeng Huang, Yongwen Ma, Jinquan Wan, Yan Wang

**Affiliations:** 1 College of Environment and Energy, South China University of Technology, Guangzhou, 510006 China; 2 The Key Lab of Pollution Control and Ecosystem Restoration in Industry Clusters, Ministry of Education, South China University of Technology, Guangzhou, 510006 China; 3 State Key Laboratory of Pulp and Paper Engineering, South China University of Technology, Guangzhou, 510641 China

**Keywords:** Environmental monitoring, Environmental impact

## Abstract

In this study, the hydrodynamic characteristic of a lab-scale internal circulation (IC) anaerobic reactor was investigated. We found that the gradual Increasing-Size Continuous Stirred-Tank Reactors (ISC) model is desirable to describe the hydraulic character of the reactor. As a generalized anaerobic digestion model, Anaerobic Digestion Model No.1 (ADM1) was combined simultaneously with the ISC model to simulate the effluent of the IC reactor. Both the stable running and overloading shock tests were carried out to validate the simulation. The mathematical simulation results agreed well with the experimental observation. This proposed model may be valuable to simulate the performance of the IC reactor effectively and to supply a useful tool to for designing and operating other anaerobic reactors.

## Introduction

Anaerobic bio-reactors have been widely employed to treat high-strength organic wastewater due to their advantages of reducing organic pollutants, producing renewable energy, yielding little residual activated sludge, and having low expenditure because they do not require aeration^1^. The Upflow Anaerobic Sludge Bed (UASB) reactor that was developed from a simple anaerobic stirred tank maintains large active biomass in a granule formation for a long period, which forms a sludge bed at the reactor under the layer^2,3^. As one of the successors to the UASB, the internal circulation (IC) reactor can be regarded as two UASB reactors stacked together. The IC reactor not only retains abundant activated granular sludge as the UASB does, but it also automatically engenders the internal circulation of sludge flow by the biogas flow within the reactor^4^. Without an external driving force, this circulation expands and fluidizes the sludge bed, which vigorously improves the contact of the active biomass and organic pollutants. Consequently, the mass transfer efficiency between the liquid-solid phases is enhanced^5,6^. The organic loading rate capacity of the IC reactor is up to 30 kg COD/(m^3^ *d), compared with 15 kg COD/(m^3^ *d) of the UASB reactor^7–9^. At present, many studies have been reported on the hydraulic character of anaerobic reactors; for example: UASB reactor, anaerobic baffled reactor, expanded granular sludge bed reactor, compartmentalized anaerobic reactor, and spiral symmetry stream anaerobic reactor, etc^10–14^. However, the hydraulic character of the IC reactor was not yet fully discussed.

Since the publication in 2002, by the International Water Association (IWA) task group, Anaerobic Digestion Model NO.1 (ADM1) has been widely applied to simulate the process of the anaerobic digestion^15,16^. This structured model includes 19 steps to describe biochemical processes, including particulates disintegration, hydrolysis, acidogenesis, acetogenesis, and methanogenesis, as well as physico-chemical equations to describe ion association/dissociation and gas-liquid transfer^10^. The ADM1 implement system set by the IWA task group was a completely stirred reactor with a single input and output stream and constant volume. The hydrodynamic of the implement system is ideal mixing^15^. Recently, most of the studies that have applied ADM1 also presumed that the hydraulic pattern of the reactor was ideal or near-ideal mixing, as was performed by the IWA task group^17–21^. However, Brucato *et al*.^22^ pointed out that the geometry of mixing tanks may generate considerable concentration gradients, and these inhomogeneities is bound to lead to uncertainties on the reaction result. Amini *et al*.^23^ ever used two- and three-phase computational fluid dynamics (CFD) simulated the full-scale Membrane Bioreactor, by analysed fluid-flow pattern, shear stress and cross-flow velocity. Based on smoothed particle hydrodynamics model which was coupled with Activated Sludge Model No. 1, Meister *et al*.^24^ developed a wastewater treatment model, which successfully computed spatial distribution of compounds in full-scale plant basins and the quantified the evolution of compounds. Hulle *et al*.^20^ pointed out that a correct description of the mixing behaviour of an anaerobic digester is necessary to accurately model and predict the reactor performance. Consequently, the simplified mixing pattern might lead to mistaken calibration of the bio-kinetic model^25^. For this reason, it is meaningful to consider the hydraulic characteristics of the reactor when ADM1 is implemented.

This study aimed to effectively simulate the effluent performance of the anaerobic reactor. A lab-scale IC reactor was set up, the hydraulic characteristics of which was investigated by the lithium-ion tracing technique. Three kinds of tank-in-series models were used to depict the hydraulic character of the reactor, and we found that the Increasing-Size Continuous Stirred-Tank Reactors (ISC) model was the most suitable. Although ADM1 was used in many previous anaerobic digestion studies, we are the first to simultaneously consider the hydraulic dynamic of the reactor when implementing ADM1. By combining it with the ISC model, the ADM1 mathematical model simulated the effluent COD (COD_*eff*_) both in steady and in over-loading states, the result of which fitted the experimental observation quite well. This mathematical model is expected to provide a beneficial method to definitely and effectively simulate the anaerobic reactor, as well as to provide several useful details on the reactor performance.

## Methods

### Synthetic wastewater

Synthetic wastewater with glucose, urea, and KH_2_PO_4_ (Shanghai Aladdin, China) was used as an organic influent substrate. The basic chemical oxygen demand (COD) concentration was 3 g/L (3 kg/m^3^), and the ratio of ${\rm{COD}}\,:\,{{\rm{NH}}}_{4}^{+}\,-\,{\rm{N}}:{{\rm{PO}}}_{4}^{3-}\,-\,{\rm{P}}$ was 200:5:1. An amount of the trace elements as inorganic nutrient were supplemented as follows (in mg/L): CaCl_2_∙2H_2_O 330, CuSO_4_∙5H_2_O 250, EDTA 5000, CoCl_2_∙6H_2_O 240, NiCl_2_∙6H_2_O 190, MnCl_2_∙4H_2_O 990, H_3_BO_4_ 14, NH_4_MoO_4_∙4H_2_O 9, ZnCl_2_ 20, FeCl_3_∙5H_2_O 250, and MgSO4 500 (Sinopharm, China). The effluent pH was maintained at 7.0 ± 0.4 by adding and adjusting the dosage of NaHCO_3_ (Sinopharm, China).

### Experimental setup and operation

**Figure 1:**
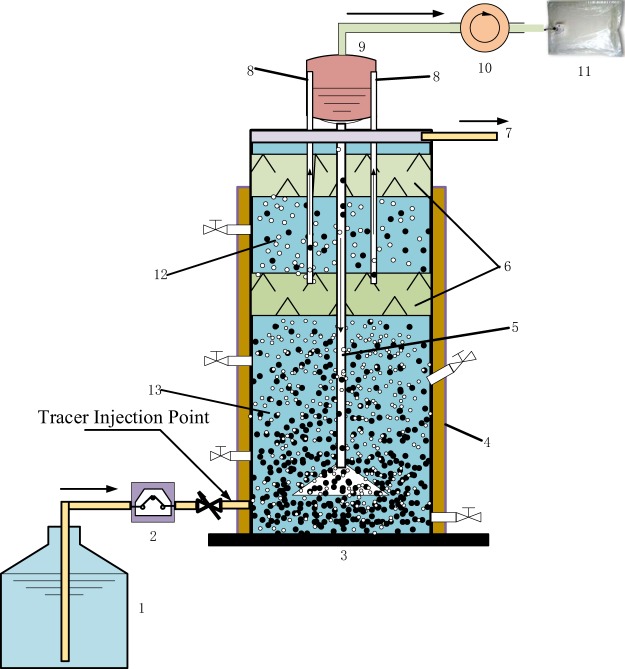
Schematic diagram of this research equipment mainly contains a synthetic wastewater tank, a lab-scale IC reactor and a gas detection device. The lab-scale IC reactor is made of Plexiglas and has a cylindrical configuration, with a total working volume of 26 L. 1. Synthetic wastewater tank; 2. Peristaltic pump; 3. IC reactor; 4. Water jacket; 5. Recirculation tube; 6. Three-phase separator; 7. Effluent water pipe; 8. Riser tubes; 9. Gas-water separation chamber; 10. Wet gas flow meter; 11. Gas sample collector; 12. Precipitation compartment; 13. Reaction compartment of expanded bed.

The lab-scale IC reactor consisted of a synthetic wastewater tank, a peristaltic pump, an IC reactor and a wet gas flow meter, as shown in Fig. 1. The reactor was made of Plexiglas and had a cylindrical configuration. The internal diameter was 200 mm, and the height of the reaction zone was 1125 mm, with a total working volume of 26 L. The ratio of the under-expanded sludge bed compartment to the upper precipitation compartment was 4:1. The reactor was equipped with a water jacket, and all of the experiments were performed under a mesospheric temperature (37 ± 2 °C).

The IC reactor was inoculated with methanogenic granule sludge from a full-scale IC anaerobic reactor that treats pulp and papermaking wastewater (Guangzhou City, China). Then, 8 L seed sludge was inoculated and sparged with pure nitrogen gas for a half hour before use. The reactor was cultivated with synthetic wastewater for one month, with a 24 h hydraulic residence time (HRT). When the COD removal rate steadily reached 80%, the reactor was considered to be in stable running condition.

### Experimental data collection

Residence time distribution (RTD) research based on the tracing technique is a common method of studying the mixing pattern of bio-chemical reactors^26^. In this study, eight tracing runs were carried out to investigate the RTD of the IC reactor at 4 different HRTs. The R1-R4 were conducted under normal operation with sludge, and the R5-R8 were conducted under the IC reactor without sludge (shown in Table 1).

**Table 1:** Operation Condition of the Tracing Experiment.

	HRT (h)	Flow Rate (L/h)	COD Loading Rate (g/L/d)	Within Biomass
R1	24	0.75	3	Yes
R2	16	1.13	4.5	Yes
R3	8	2.25	12	Yes
R4	4	4.50	18	Yes
R5	24	1.08	0	No
R6	16	1.62	0	No
R7	8	3.24	0	No
R8	4	6.48	0	No

It has been reported that with lithium as the tracer, lithium-ion (Li^+^) does not adsorb onto the sludge and is not harmful to the biomass^14^. By using an injector, a solution containing 2.397 g Li_2_SO_4_∙H_2_O (260 mg Li^+^, Shanghai Aladdin, China) was injected instantaneously to produce 10 mg/L mean concentration Li^+^ in the reactor. The pulse injection (less than 2 second)^24^ was conducted at t = 0, which was exploited to obtain the exit-age function *E(t)* of lithium-ion (represented as the substrate)^27^. Samples were collected from the effluent pipe every 0.5 hour for 4 h and 8 h HRTs, and every 1 hour for 16 h and 24 h HRTs. Samples were centrifuged and filtered before analysis^16^. The lithium-ion concentration was detected by an inductive coupled plasma emission spectrometer (ICP, PerkinElmer-Optima 8300, US) at a wavelength of 670.8 nm, according to the Standard Methods^28^.

Effluent water COD was determined using the potassium dichromate method^29^. The concentration of volatile fatty acids (VFA) was measured by a gas chromatograph external standard method. To inhibit the adsorption of organic acids by using a chromatographic column, all of the effluent samples were pre-treated by using a high-speed refrigerated centrifuge and a microporous membrane filtrate. The operating condition of the chromatographic detection was set as: the injector and hydrogen flame ion detector (FID) temperatures were 200 °C and 300 °C, respectively; nitrogen was used as the carrier gas under a 5 mL/min flow rate; and a DB-FFAP (30 m × 0.32 mm × 0.5 µm, Agilent, China) capillary column was used. The configuration of the column operating temperature was originally 80 °C holding for 2 minutes, then increased to 200 °C at the rate of 10 °C/min and held for 5 minutes before running detection.

### Theoretical interpretation

#### Normalized RTD curves

The hydraulic characteristics of the IC reactor were determined based on RTD curves. The curves were obtained from tracer studies. The RTD exit-age distribution function *E(t)*, the mean residence time $\overline{t}$ and the distribution variance of the RTD ${\sigma }_{t}^{2}$ are calculated as follows^12^:

1 $$E(t)=\frac{C(t)}{{\int }_{0}^{\infty }C(t)dt}$$

2 $$\overline{t}=\frac{{\int }_{0}^{\infty }tE(t)dt}{{\int }_{0}^{\infty }E(t)dt}={\int }_{0}^{\infty }tE(t)dt$$

3 $${\sigma }_{t}^{2}=\frac{{\int }_{0}^{\infty }{(t-\overline{t})}^{2}E(t)dt}{{\int }_{0}^{\infty }E(t)dt}={\int }_{0}^{\infty }{t}^{2}E(t)dt-{(\overline{t})}^{2}$$

where *t* is time, h; and *C(t)* is the tracer concentration at time *t*, mg/L.

To compare the flow patterns at different HRTs, Eqs (1–3) were normalized by the mean residence time $\overline{t}$. The normalized time *θ*, normalized RTD exit-age distribution function *E(θ)* and the dimensionless variance ${\sigma }_{\theta }^{2}$ are described as follows:

4 $$\theta =\frac{t}{\overline{t}}$$

5 $$E(\theta )=\overline{t}\times E(t)$$

6 $${\sigma }_{\theta }^{2}=\frac{{\sigma }_{t}^{2}}{\overline{t}}$$

#### Dispersion number & Péclet number

Theoretically, when the back-mixing in the reactor is weak, the axial dispersion plug flow (PF) model can be applied to simulate the hydrodynamic of the reactor. The dispersion number *D/υL* (dimensionless) is used to characterize the back-mixing intensity of the reactor system. The Péclet number (*Pe*, dimensionless) is the inverse of the dispersion number D/υL. *Pe* can be estimated as follows:

7 $${\sigma }_{\theta }^{2}=\frac{2}{Pe}-\frac{2}{P{e}^{2}}(1-{e}^{-Pe})$$

An ideal totally mixed reactor will show *Pe* = 1, while the ideal PF will show *Pe* = ∞ at the other extreme^14^.

#### Tank-in-series (TIS) models

**Figure 2:**
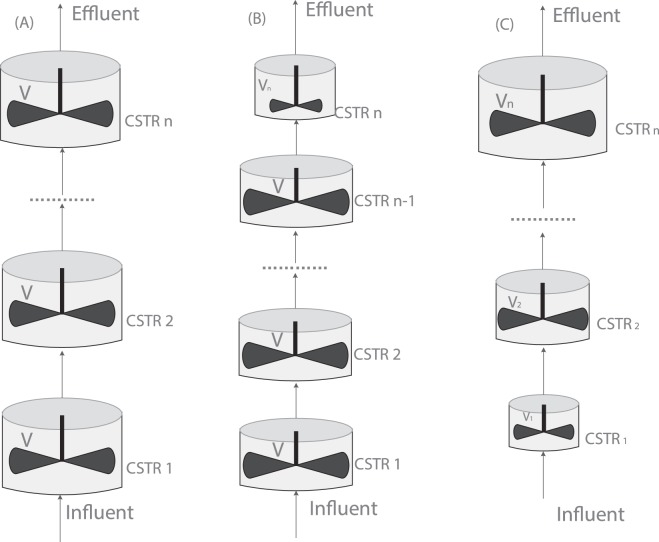
Schematic diagram of multi-CSTRs (continual stirred tank reactor) tank-in-series simulated models: (**A**) equal-sized CSTRs (ESC) model; (**B**) extended equal-sized CSTRs (EESC) model; (**C**) increasing-size CSTRs (ISC) model.

As a non-ideal flow, the hydrodynamics of the anaerobic reactor can be well described by the multi-CSTRs (continuous stirred tank reactor) tank-in-series model^25,29^. Figure 2 shows these models.

The equal-sized CSTRs model (ESC) is commonly used, which is described by the Erlang distribution:

8 $$E(\theta )=\frac{{N}^{N}}{(N-1)!}{\theta }^{N-1}{e}^{-N\theta }$$

The number N value (N_ESC_) of ESC in Eq. (8) can be counted as follows:

9 $$N=\frac{1}{{\sigma }_{\theta }^{2}}$$

If N_ESC_ is a non-integer, N_ESC_ needs to be rounded to the nearest integer number. Furthermore, an extended equal-sized CSTRs (EESC) model can be used by the subset of the gamma distribution^27,30^, while the N value (N_EESC_) is a non-integer in the equation, as follows:

10 $$E(\theta )=\frac{{N}^{N}}{{\rm{\Gamma }}(N)}{\theta }^{N-1}{e}^{-N\theta }$$

The previous research studies have illustrated that the distribution of the substrates and intermediates along the axis of the reactor were far different, and the increasing-size CSTRs model (ISC) would perform better at simulating the performance of the reactor^30,31^. The ISC model can be expressed by an equation set as below^19^:

11 $$\{\begin{array}{c}E(\theta )=\sum _{i=1}^{N}\,[\frac{{r}_{i}^{N-2}}{\underset{j=1,j\ne i}{\overset{N}{{\rm{\Pi }}}}({r}_{i}+{r}_{j})}{e}^{-\frac{\theta }{{r}_{i}}}]\\ {r}_{1}+{r}_{2}+\ldots +{r}_{N}=1\\ \frac{{r}_{k}}{{r}_{k-1}} > 1,2\le k\le N\end{array}$$

where *r*_*i*_ is the volume fraction coefficient and N is the number of tanks.

#### ADM1 implement

Based on the hydraulic calculation result, AMD1 was used to simulate the effluent of the lab-scale IC reactor by AQUASIM2.0 (published by EAWAG)^32^. The biological degradation processes are described by substrate uptake Monod-type kinetics equations, while other extracellular processes (e.g., disintegration and hydrolysis) and biomass decay are described by first-order kinetics equations^33^. Using a CSTR model with a single input and output stream and constant liquid volume, the IWA Task Group has provided the differential equation of the mass balance for each state component in the liquid phase, as follows^15^:

12 $$\frac{d{S}_{liq,i}}{dt}=\frac{{q}_{in}{S}_{in,i}}{{V}_{liq}}-\frac{{q}_{out}{S}_{liq,i}}{{V}_{liq}}+\sum _{j=1-19}{\rho }_{j}{\nu }_{i,j}$$

Despite all parameters in the equations theoretically affecting the outcome of the model, the sensitivity degree of the parameters was far different. In particular, most of the parameters have low sensitivity. A sensitivity analysis was used to identify the dominant parameters, which was conducted by employing the absolute-relative sensitivity function supported by AQUASIM2.0. The function is calculated as follows^32^:

13 $${\delta }_{y,p}^{a,r}=\frac{1}{y}\frac{\partial y}{\partial p}$$

where *y* is an arbitrary variable that is calculated, and *p* is a model parameter. The absolute-relative sensitivity function measures the absolute change in *y* for a 100% change in *p*. Their units do not depend on the unit of the parameter, which makes quantitative comparisons of the effect of different parameters *p* on the same variable *y* possible.

The model parameters that are represented by constant variables can be estimated by minimizing the sum of the squares of the weighted deviations between the measurements and calculating the results of the model as follows:

14 $${\chi }^{2}=\sum _{i=1}^{n}{(\frac{{y}_{meas,i}-{y}_{i}}{{\sigma }_{meas,i}})}^{2}$$

where *y*_*meas,i*_ is the *i-th* measurement; *σ*_*meas,i*_ is its standard deviation; and *y*_*i*_ is the calculated value of the model.

Using the algorithm provided the by AQUASIM2.0, both the sensitivity analysis and the model parameters were calculated.

## Results and Discussion

### HRT distribution in the trace experiment

**Figure 3:**
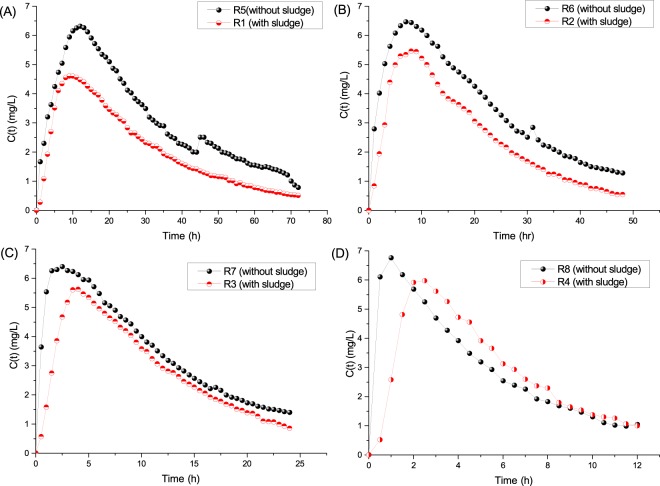
Schematic diagram of the tracer concentration at time t (*C(t)-t* curve). In each diagram, the black dot plot depicts the *C(t)-t* curve under blank operation without sludge, and the red dot depicts the *C(t)-t* curve under normal operation with sludge. HRT of diagrams (**A–D**) are 24 h, 16 h, 8 h and 4 h, respectively.

**Figure 4:**
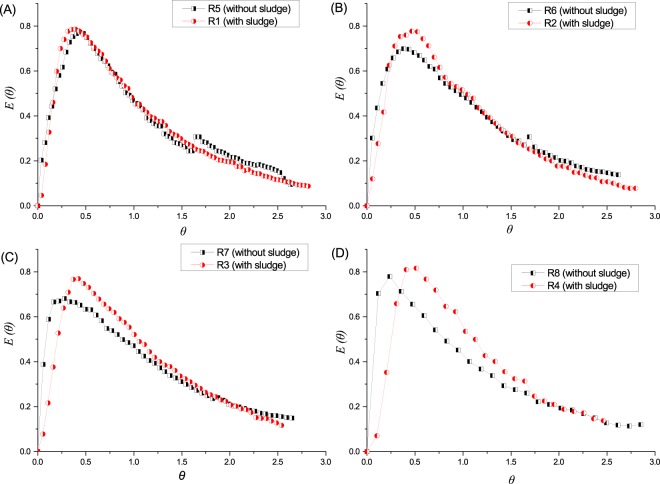
Schematic diagram of the normalized exit-age function *E(θ)* versus the normalized time θ (*E(θ)-θ* curve). In each diagram, the black dot plot depicts the *E(θ)-θ* curve under blank operation without sludge, and the red dot depicts the *E(θ)-θ* curve under normal operation with sludge. HRT of diagrams (**A–D**) are 24 h, 16 h, 8 h and 4 h, respectively.

Using the data collected from the R1-R8 tracing experiment, we obtained the HRT distributive information *C(t)-t* curves (Fig. 3), which were converted to *E(θ)-θ* curves (Fig. 4)^12,21^. By comparing the *C(t)-t* curves of the tracing experiment at different runs, we found that the *C(t)-t* Li^+^ peak value intensity of normal operation was lower than that of the no sludge operation, which confirmed the findings of a previous tracing study^12^. Nevertheless, when the *C(t)-t* curves were normalized, and the *E(θ)-θ* peak value intensity of the with-sludge operation was obviously higher than that of counterparts without sludge, which reflected that the RTD in normal operation was intensive and the flow pattern tended to be PF^14^.

From the *C(t)-t* curves or *E(θ)-θ* curves, the peak value occurrence time (PVOT) of the normal operation was later than that without the sludge operation on a short HRT (comparing R4 and R8). Along with the extended HRT, the PVOT of the two operations were close (comparing R3 and R7, R2 and R6, and R1 and R5). These results illustrated that: the reactor structure fundamentally determined the RTD of the substrates; the sludge can detain the substrates, which showed apparently at the short HRT; nevertheless, the sludge detaining effect would degrade along with the extension of HRT, which might be attributed to the perturbation effect of generating bubbles in the reactor.

### Back-mixing degree and degradation efficiency

Back-mixing refers to the mixing degree of substrates in the reactor, which exerts an important effect on pollutant removal^12^. The back-mixing degree can be described by N or *D/υL*^34^. Table 2 shows the RTD analyses results (*D/υL*, *Pe* and TIS model tank number N value) under all experiment conditions. From Eq. (9), the calculated tank number N value is able to identify the mixing pattern. If N tends to be 1, the reactor approximates to the CSTR (totally mixed); on the contrary, when N tends towards ∞, the reactor approximates to the PF reactor (without mixing)^34^. According to Tomlinson *et al*.^35^, *D/υL* = 0.02 (*Pe* = 50) is defined as the intermediate degree of dispersion, and *D/υL* ≥ 0.2 (*Pe* ≤ 5) is defined as large dispersion.

**Table 2:** Results of the RTD analyses under all conditions.

	HRT (h)	D/υL	Pe	N value	Within Biomass
R1	24	0.32	3.12	2.25	Yes
R2	16	0.29	3.47	2.41	Yes
R3	8	0.25	4.02	2.66	Yes
R4	4	0.21	4.74	2.99	Yes
R5	24	0.31	3.19	2.28	No
R6	16	0.31	3.27	2.32	No
R7	8	0.35	2.89	2.15	No
R8	4	0.42	2.40	1.93	No

The Run5–8 were carried out without sludge, and the N values were 2.28, 2.32, 2.15, and 1.93 under the HRT, from 24 to 4, respectively, which revealed that the mixing pattern tends to be completely mixed, and the back-mixing degree was increased with the decreasing HRT. The dispersion number D/υL values (0.31, 0.31, 0.35, and 0.42, respectively) were greater than 0.02. Therefore, the dispersion of the reactor was large and the back-mixing was strong. The Run1–4 were executed under normal conditions (with sludge), and the N values were 2.25, 2.41, 2.66 and 2.99, respectively, under HRT from 24 h to 4 h, which implied that the mixing pattern also tends to be strongly mixed, but the back-mixing degree decreased with the decreasing HRT. The dispersion numbers D/υL (0.32, 0.29, 0.25 and 0.21, respectively) were still greater than 0.02 but were apparently less than the value of the counterparts without sludge. The result illustrated that the configuration of the reactor mainly determines the mixing pattern. Within the biomass, the granule sludge and the generation of bubbles produce a coaction to stimulate the mixing pattern to be PF. This effect was obvious under a short HRT.

Theoretically, the pollutant removal rate in the absolute PF pattern is higher than the rate in the absolute CSTR pattern. The calculated tank number N values under the normal experiment were from 2.25 to 2.99 (HRT from 24 to 4) and were no more than 3, which indicated that this IC reactor has a moderate back-mixing pattern between the PF and the completely mixed pattern^34^. With the decreasing HRT, the mixing pattern tends to be a PF pattern. Generally, when the reactor is dominated under Monod or first-order biological kinetics, a PF pattern will be more effective^36^. Consequently, if the anaerobic bioreactor has an absolute PF pattern, the production of VFAs will accumulate at the bottom layer and the pH will descend there; at the same time, the methane production occurs at only the top layer and the pH value will ascend^12^. It should be noted that methanogenic bacteria may be inhibited by a low pH, when the pH of the reaction is less than 6.5^37,38^. This adverse result can break down the methane production reaction.

In contrast, moderate back-mixing can produce a continuous stirring between the liquid-solid phases, which not only enhances the transfer action on pollutants and granule sludge but also increases the balance of substrates, the pH, and nutrients in the reactor. Therefore, the moderate back-mixing effect may be beneficial for the highly efficient degradation capacity of IC reactor.

### TIS model simulation

Through minimizing the sum of the squared difference χ^2^ between the calculative values and the experimental values of the effluent samples^11,30^ on normal operation, the TIS model simulation parameters were estimated and are listed in Table 3.

**Table 3:** Parameter Estimation Results for TIS Models.

TIS models	Parameter	Runs on normal operation			
		R1 24 hr	R2 16 hr	R3 8 hr	R4 4 hr
ESC	N value (N_ESC_)	2	2	3	3
	Final χ^2^	14.38	5.95	43.61	14.14
EESC	N value (N_EESC_)	2.25	2.41	2.66	2.99
	Tanks volume ratio	1:1:0.25	1:1:0.41	1:1:0.66	1:1:0.99
	Final χ^2^	26.11	15.03	26.19	14.08
ISC	N value (N_ISC_)	3	3	3	3
	Tanks volume ratio	1:5:20	1:15:37	1:15:18	1:1.2:5
	Final χ^2^	8.77	4.92	9.52	4.34

The number of tanks for the EESC model (N_EESC_ value) was counted by the tracing data using Eq. (9), and the N_EESC_ values were 2.25, 2.41, 2.66 and 2.99 for normal operation at an HRT of 24, 16, 8 and 4 h, respectively. Then, the number of tanks for the ESC model (N_ESC_ value) was rounded from those of EESC to the nearest integer number, and the N_ESC_ values were 2, 2, 3 and 3 for normal operation at 4 different HRTs. The estimated number of tanks for the ISC model (N_ISC_ value) was 3 at all HRTs.

**Figure 5:**
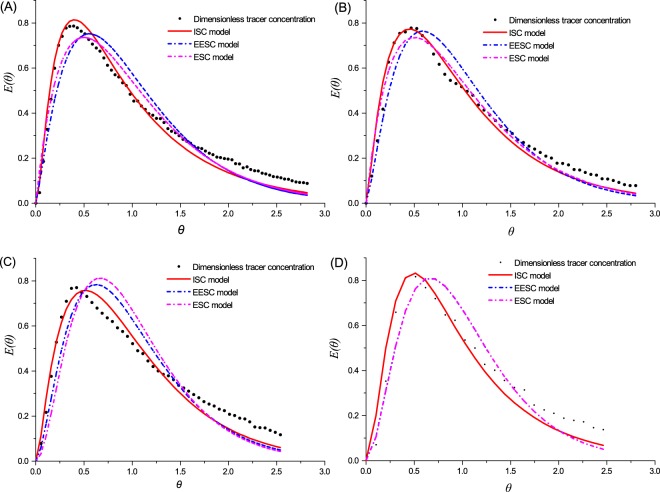
Schematic diagram of three kinds of tank-in-series (TIS) models of the simulation result. In each diagram, black dots represent the measured tracer concentration; the red line represent the increasing-size CSTRs (ISC) model simulation; the blue dot represents the extended equal-sized CSTRs (EESC) model; the light purple dotted-line represents the equal-sized CSTRs (ESC) model. HRT of the diagrams (**A–D**) are 24 h, 16 h, 8 h and 4 h, respectively.

Figure 5 shows the simulated results of the three models. It is easy to find that the accuracy of the ISC model is higher than that of the other two models. This finding agreed with the research results of Dai *et al*.^11^ and Ren *et al*.^30^, who even pointed out that it was convenient to modify the ISC model to accurately simulate the hydrodynamic of the anaerobic bioreactor. In this study, the estimated N_ISC_ values were acquired according to Eq. (9) calculation results, and the tank volume ratio was further estimated by minimizing the squared difference χ^2^. Therefore, exploiting the ISC model would easily improve the simulation results.

### Sensitivity analysis of the ADM1 parameter

ADM1 involves 19 biochemical processes and 26 component variables^15^. All of the biochemical process rates are described by Monod uptake equations or by first-order kinetic equations^39^. Despite the fact that all of the parameters in the equations theoretically affect the output of the model, the sensitivity degree of the parameters may be far different from one to another, and many parameters have little impact. It is necessary to analyse and distinguish the important parameters which have the significant impact on the model calculation. Chen *et al*.^34^ ever pointed out that sensitivity analysis would help to identify the important parameters and reduces the complexity of parameter tuning, who only chose *k*_*m*_ parameters (specific Monod maximum uptake rate) of propionate and acetate for parameter tuning. Barrera *et al*.^33^ employed local relative sensitivity analysis method to calculate sensitivity functions for the dynamic simulations. As the most sensitive parameters, *k*_*m*_ parameters of propionate, acetate, hydrogen, and Yield of hydrogen were used for calibration in the anaerobic digestion with sulphate reduction of cane-molasses vinasse. The sensitivity analysis was a useful method to identify the dominant parameters, to reduce the model complexity and to determine the main processes^40,41^.

In the research, lab synthetic wastewater was used, and the glucose was the only substrate in the influent. Glucose, as a simple monosaccharide, undergoes easy uptake by sugar degraders (X_su_) and will be decomposed directly into VFAs (butyrate, propionate, and acetate, etc.) and H_2_^15,40^. The VFAs are the main ingredients of the effluent COD (COD_*eff*_). Thus, to accurately predict the COD_*eff*_, the kinetic parameters of the uptake process rates of butyrate, propionate and acetate were used in the calculation.

**Figure 6:**
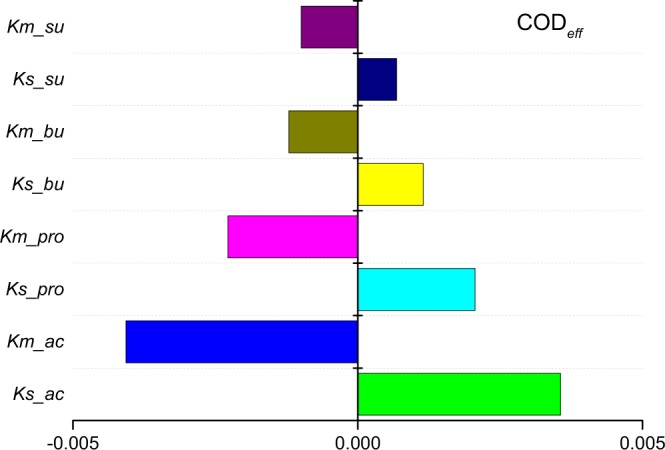
Relative model parameters of the sensitivity analysis accounting for COD_*eff*_. The absolute-relative sensitivity analysis of the *k*_*m*_ parameter (specific Monod maximum uptake rate) and the *K*_*s*_ parameter (Monod half saturation constant) relating to sugar, butyrate, propionate, and acetate degrading are depicted.

**Figure 7:**
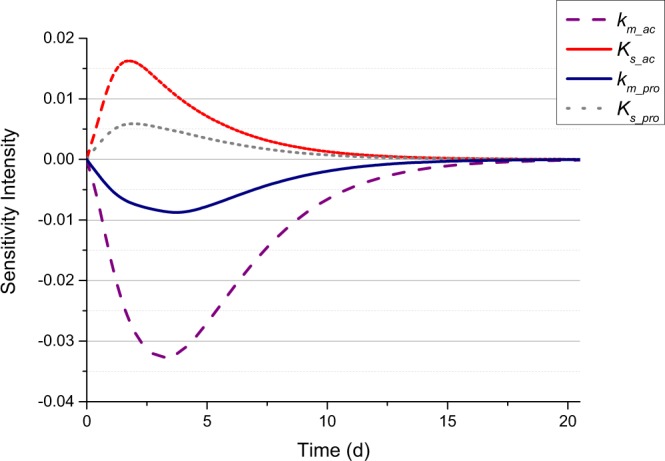
Parameter sensitivity analysis of *k*_*m*_ & *K*_*s*_ for acetate and propionate by ADM1 incorporated with the ISC hydraulic model. The red dotted line and purple dots represent the *k*_*m*_ & *K*_*s*_ of acetate, respectively, and the grey dots and black line represent those of the propionate, respectively.

Using ADM1 incorporated with the ISC hydraulic model, a sensitivity analysis of the parameters was executed by AQUASIM. The absolute-relative sensitivity analysis of the *k*_*m*_ parameter (specific Monod maximum uptake rate) and the *K*_*s*_ parameter (Monod half saturation constant) relating to these ingredients are shown in Fig. 6. The sensitivity of *k*_*m*_ in the process rate equation is higher than that of *K*_*s*_ in the same process rate equation. The *k*_*m*_ and *K*_*s*_ values of the uptake process in the acetate and propionate uptake processes showed higher sensitivity than their counterparts in the butyrate and sugar uptake processes, especially in the *k*_*m*_ value of the acetate uptake process. Therefore, these four parameters were used for parameter adjustments, which would be considered in the next step of the mathematical simulation. The sensitivity curves of both *k*_*m*_ and *K*_*s*_ parameters for acetate and propionate are shown in Fig. 7.

### Mathematical model development and validation

Referring to the parameter estimation results for the ISC hydraulic model (Table 3), we chose the medium ratio value 1:2:5 for the developed ISC model (a 2 litre tank, 4 litre tank and 10 litre tank were joined together) to simulate the hydrodynamic of IC reactor. Then, ADM1 incorporated with this ISC hydraulic model were set as the simulation mathematical model.

Anaerobic reactor system was sensitive to influent fluctuation, such as high organic loading shock, hydraulic shock, and even toxic wastes shock etc. The disturbances might adversely affect the quality of effluent what should be monitored and predicted earlier. Consequently, to validate the mathematical model in wastewater COD_*eff*_ prediction, both the stable running test and overloading shock test were carried out in our experiments. On table running test, the influence was maintain the basic COD concentration (3 g/L), and the HRT was maintain 24 h; on hydraulic shock test, the influence was six times flow rate lasting 12 hours, with basic COD concentration; and on the organic loading shock, the influence was six times COD concentration (18 g/L) lasting 8 hours, with 24 h HRT.

According to the outcome of the sensitivity analysis result, we adjusted the *k*_m_ac_, *k*_m_pro_, *K*_s_ac_ and *K*_s_pro_ values in this mathematical model simulation. Other parameters were adopted from the recommended values of the IWA task group^15^. These adjusted values are listed in Table 4.

**Table 4:** Parameter Estimation Results of the ISC Model.

*k*_m_ac_	*k*_m_pro_	*K*_s_ac_	*K*_s_pro_	*χ* ^2^
13.13	9.90	0.14	0.30	0.469

**Figure 8:**
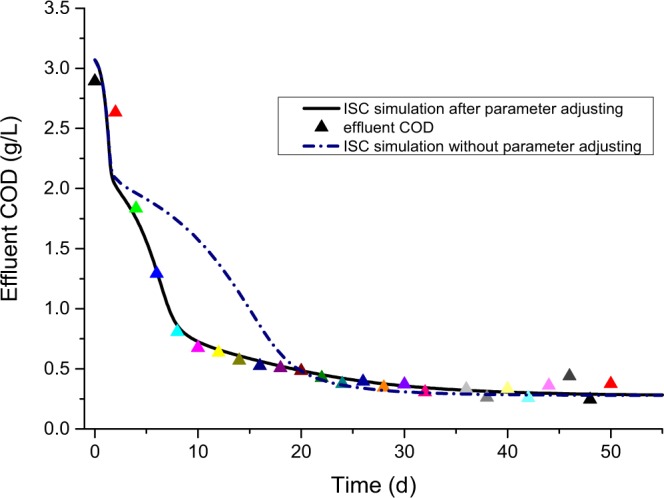
Simulation result of the IC reactor start-up on stable running by ADM1-based ISC model, influent COD and HRT of which was 3 g/L and 24 hours, respectively. The blue dotted-line represents the simulation result with the original parameter, and the black line represents the simulation with the adjusting maximum uptake rate and half saturation constant.

The simulation result of the IC reactor start-up was on stable running, when the influent COD concentration was maintained at 3 g/L and HRT at 24 h (shown in Fig. 8). After adjusting the maximum uptake rate and the half saturation constant, the simulation COD effluent agreed with the experimental effluent COD. Comparatively, the simulation with the original specific Monod maximum uptake rate and Monod half saturation constant, did not fit the experimental result well, especially at the initial stage before the reactor system was running stably ( < 20 d).

**Figure 9:**
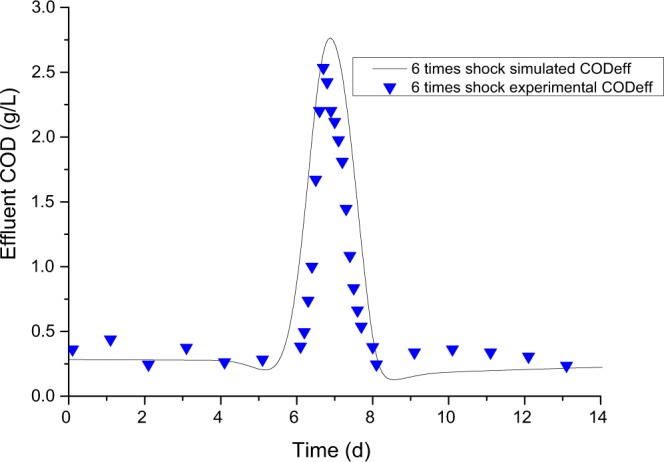
Simulation result of the IC reactor under six times organic loading shock, which lasted 8 h.

**Figure 10:**
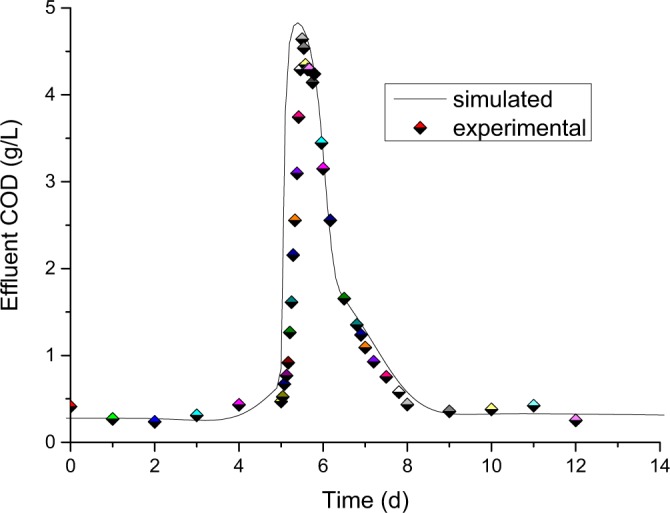
Simulation result of the IC reactor under 6 times hydraulic shock, which lasted 12 hours.

Short-time overloading, such as a huge pollutant influent or water flow shock, are common in industrial wastewater treatments^42,43^. Based on regular loading simulation, we further simulated the over-organic and over-hydraulic shock tests. Organic loading shock lasting 8 hours was carried out six times, the result of which is shown in Fig. 9. The simulation COD_*eff*_ matched quite well with the experimental COD_*eff*_. Over-hydraulic shock lasting 12 hours was also conducted six times. As shown in Fig. 10, the simulation result agreed with the hydraulic shock experimental COD_*eff*_. Both of these well modelled results validated that the mathematical model of ADM1 incorporated with the ISC hydraulic model can predict well the IC reactor effluent COD.

## Conclusion

A lab-scale internal circulation (IC) anaerobic reactor (26 L) was set up, and the hydrodynamic characteristics of this IC reactor were studied. Three kinds of tank-in-series models were used to simulate the hydrodynamics of this reactor, and the gradually increasing-size CSTRs model (ISC model) was more suitable to fit the hydraulic character than the other two models. Based on the calculations of RTD dimensionless variance, 3 tanks in this estimate had been used by the TIS model. We chose the medium ratio value 1:2:5 of the ISC model to simulate the hydraulic dynamic of this reactor. Based on this, the ADM1 was combined with the ISC model to predict the effluent COD. Both stable running and overloading shock tests were executed, and the simulation results agreed with the experimental data. The proposed model in this research might be valuable to monitor reactor effluent effectively and to supply a useful tool to design and operate a full-scale anaerobic reactor.

## References

[1] Li S, Nan J, Gao F (2016). Hydraulic characteristics and performance modeling of a modified anaerobic baffled reactor (MABR). Chemical Engineering Journal.

[2] Tao B (2017). Enhancement of microbial density and methane production in advanced anaerobic digestion of secondary sewage sludge by continuous removal of ammonia. Bioresource Technology.

[3] Kamali M, Gameiro T, Costa MEV, Capela I (2016). Anaerobic digestion of pulp and paper mill wastes–An overview of the developments and improvement opportunities. Chemical Engineering Journal.

[4] Macarie H (2018). Strategy to identify the causes and to solve a sludge granulation problem in methanogenic reactors: application to a full-scale plant treating cheese wastewater. Environmental science and pollution research international.

[5] Wang, Y., Liu, X., Zhuang, W., Zhou, J. & Wang, J. In 2011 International Conference on Materials for Renewable Energy & Environment. 423–427 (2011).

[6] Mao C, Feng Y, Wang X, Ren G (2015). Review on research achievements of biogas from anaerobic digestion. Renewable and Sustainable Energy Reviews.

[7] Karadag D (2015). Anaerobic granular reactors for the treatment of dairy wastewater: A review. International Journal of Dairy Technology.

[8] Rajagopal R, Torrijos M, Kumar P, Mehrotra I (2013). Substrate removal kinetics in high-rate upflow anaerobic filters packed with low-density polyethylene media treating high-strength agro-food wastewaters. Journal of Environmental Management.

[9] Hamza RA, Iorhemen OT, Tay JH (2016). Advances in biological systems for the treatment of high-strength wastewater. Journal of Water Process Engineering.

[10] Batstone DJ, Puyol D, Flores-Alsina X, Rodríguez J (2015). Mathematical modelling of anaerobic digestion processes: applications and future needs. Reviews in Environmental Science and Bio/Technology.

[11] Dai R (2016). Dispersion characteristics of a spiral symmetry stream anaerobic bio-reactor. Biochemical Engineering Journal.

[12] Ji J-y, Zheng K, Xing Y-j, Zheng P (2012). Hydraulic characteristics and their effects on working performance of compartmentalized anaerobic reactor. Bioresource Technology.

[13] Qi W-K, Hojo T, Li Y-Y (2013). Hydraulic characteristics simulation of an innovative self-agitation anaerobic baffled reactor (SA-ABR). Bioresource Technology.

[14] Sarathai Y, Koottatep T, Morel A (2010). Hydraulic characteristics of an anaerobic baffled reactor as onsite wastewater treatment system. Journal of Environmental Sciences.

[15] Batstone DJ (2002). The IWA anaerobic digestion model no 1 (ADM1). Water Science and Technology.

[16] Yu, L., Wensel, P. C., Ma, J. & Chen, S. Mathematical modeling in anaerobic digestion (AD). *Journal of Bioremediation and Biodegradation* 5 (2014).

[17] Delrue F (2010). Modelling a full scale membrane bioreactor using Activated Sludge Model No.1: challenges and solutions. Water Science and Technology.

[18] Naessens, W., Maere, T. & Nopens, I. Critical review of membrane bioreactor models – Part 1:Biokinetic and filtration models. Bioresource Technology 122, 95–106 10. 1016/j.biortech.2012.05.070 (2012). 10.1016/j.biortech.2012.05.070 22721681

[19] Yu L, Zhao Q, Ma J, Frear C, Shulin C (2012). Experimental and modeling study of a two-stage pilot scale high solid anaerobic digester system. Bioresource Technology.

[20] Van Hulle SWH, Vesvikar M, Poutiainen H, Nopens I (2014). Importance of scale and hydrodynamics for modeling anaerobic digester performance. Chemical Engineering Journal.

[21] Chen Y (2015). Mathematical modeling of upflow anaerobic sludge blanket (UASB) reactors: Simultaneous accounting for hydrodynamics and bio-dynamics. Chemical Engineering Science.

[22] Brucato A, Ciofalo M, Grisafi F, Tocco R (2000). On the simulation of stirred tank reactors via computational fluid dynamics. Chemical Engineering Science.

[23] Amini E, Mehrnia MR, Mousavi SM, Mostoufi N (2013). Experimental Study and Computational Fluid Dynamics Simulation of a Full-Scale Membrane Bioreactor for Municipal Wastewater Treatment Application. Industrial & Engineering Chemistry Research.

[24] Meister M, Winkler D, Rezavand M, Rauch W (2017). Integrating hydrodynamics and biokinetics in wastewater treatment modelling by using smoothed particle hydrodynamics. Computers & Chemical Engineering.

[25] Naessens W, Maere T, Ratkovich N, Vedantam S, Nopens I (2012). Critical review of membrane bioreactor models–Part 2: Hydrodynamic and integrated models. Bioresource technology.

[26] Djatkov D, Effenberger M, Martinov M (2014). Method for assessing and improving the efficiency of agricultural biogas plants based on fuzzy logic and expert systems. Applied Energy.

[27] Martin AD (2000). Interpretation of residence time distribution data. Chemical Engineering Science.

[28] Rand, M., Greemberg, A. G. & Taraj, M. J. Standard Methods for Examination of Water and Waste Water (1998).

[29] Standardization, I. O. f. Water quality—Determination of the chemical oxygen demand. ISO 6060:1989 (1989).

[30] Ren TT, Mu Y, Ni BJ, Yu HQ (2009). Hydrodynamics of upflow anaerobic sludge blanket reactors. AIChE Journal.

[31] Lim SJ, Kim T-H (2014). Applicability and trends of anaerobic granular sludge treatment processes. Biomass and bioenergy.

[32] Reichert Peter (1994). AQUASIM – A TOOL FOR SIMULATION AND DATA ANALYSIS OF AQUATIC SYSTEMS. Water Science and Technology.

[33] Barrera EL (2015). Modeling the anaerobic digestion of cane-molasses vinasse: extension of the Anaerobic Digestion Model No. 1 (ADM1) with sulfate reduction for a very high strength and sulfate rich wastewater. Water research.

[34] Chen XG (2010). Flow patterns of super-high-rate anaerobic bioreactor. Bioresource Technology.

[35] Tomlinson, E. J. & Chambers, B. Effect of longitudinal mixing on the settleability of activated sludge. WRC technical report. TR 122 (1979).

[36] Donoso-Bravo A (2011). Model selection, identification and validation in anaerobic digestion: a review. Water research.

[37] Lier JBV, Zee FPVD, Frijters CTMJ, Ersahin ME (2015). Celebrating 40 years anaerobic sludge bed reactors for industrial wastewater treatment. Reviews in Environmental Science and Bio/Technology.

[38] Chen Y, Cheng JJ, Creamer KS (2008). Inhibition of anaerobic digestion process: A review. Bioresource Technology.

[39] Rosen, C. & Jeppsson, U. Aspects on ADM1 Implementation within the BSM2 Framework. Department of Industrial Electrical Engineering and Automation, Lund University, Lund, Sweden, 1–35 (2006).

[40] Dereli̇ RK, Ersahi̇n ME, Ozgun H, Ozturk I, Aydi̇n AF (2010). Applicability of Anaerobic Digestion Model No. 1 (ADM1) for a specific industrial wastewater: opium alkaloid effluents. Chemical Engineering Journal.

[41] Bernard O, Hadj-Sadok Z, Dochain D, Genovesi A, Steyer JP (2010). Dynamical model development and parameter identification for an anaerobic wastewater treatment process. Biotechnology & Bioengineering.

[42] Wan J (2011). Prediction of effluent quality of a paper mill wastewater treatment using an adaptive network-based fuzzy inference system. Applied Soft Computing.

[43] Ruan J, Chen X, Huang M, Zhang T (2016). Application of fuzzy neural networks for modeling of biodegradation and biogas production in a full-scale internal circulation anaerobic reactor. Journal of Environmental Science and Health, Part A.

